# Peripheral Artery Disease Ultrasound Assessment in Predicting the Severity of Coronary Artery Disease

**DOI:** 10.3390/life14030333

**Published:** 2024-03-01

**Authors:** Maria Olinic, Florin-Leontin Lazar, Horea-Laurentiu Onea, Calin Homorodean, Mihai Ober, Dan Tataru, Mihail Spinu, Alexandru Achim, Dan-Mircea Olinic

**Affiliations:** 1 Department of Internal Medicine, Medical Clinic No.1, Iuliu Hatieganu University of Medicine and Pharmacy, 400347 Cluj-Napoca, Romania; maria_olinic@yahoo.com (M.O.); chomorodean@yahoo.com (C.H.);; 2 Second Cardiology Department, County Emergency Hospital Cluj-Napoca, 400347 Cluj-Napoca, Romania; 3 Niculae Stancioiu Heart Institute Cluj-Napoca, 400001 Cluj-Napoca, Romania

**Keywords:** Doppler ultrasound, peripheral artery disease, coronary artery disease

## Abstract

Atherosclerosis in a progressive disease that is systemic in nature, and hence the simultaneous presentation of coronary artery disease (CAD) and peripheral artery disease (PAD) is not uncommon. As clinically manifested PAD is associated with worse cardiovascular outcomes, the timely identification of subclinical atherosclerosis seems of utmost importance. Ultrasonography (US) is an ideal imaging modality for assessing PAD that is easy to use, accurate, widely available and avoids unnecessary exposure to radiation. Several US parameters have been proposed in the assessment of PAD, with varying prognostic usefulness, depending on disease location. The aim of this review is to summarize the most important evidence available on the association between US-detected atherosclerosis in different vascular sites and the presence and severity of CAD, as well as the impact of the early detection of PAD on the outcomes of patients presenting with CAD.

## 1. Introduction 

The association of peripheral artery disease (PAD) and coronary artery disease (CAD) is not uncommon and moreover has been correlated with short- and mid-term morbidity and mortality in a series of studies [1,2,3]. Several hypotheses have been proposed to explain the worse outcomes of patients with clinical and subclinical PAD compared to those without: (1) the increased atherosclerotic burden in PAD patients [4]; (2) the higher prevalence of left main and multivessel coronary artery disease in these patients [5]; (3) the higher levels of C-reactive protein, homocysteine or amyloid A, as markers of inflammation and (4) the rates of poorly controlled diabetes and time-dependent tobacco exposure, which are significantly higher in PAD patients [6,7].

However, while there is robust evidence regarding the negative impact of clinically manifested peripheral artery disease, data regarding the impact of subclinical PAD on the outcomes of patients with CAD are still scarce. Subclinical atherosclerosis can easily be assessed by using high-resolution ultrasound, through which asymptomatic patients can be classified into subgroups of different risk for cardiovascular (CV) events according to the degree and extent of subclinical atherosclerotic lesions, as follows: normal, intima-media granulation, plaque without hemodynamic disturbance and stenotic plaque [8]. 

Despite sharing common major risk factors for atherosclerosis, the impact of different localizations of PAD and the available data for each of them differ. As subclinical atherosclerosis is often generalized, its detection remains challenging for different sites, but ultrasonography is an efficient tool, as the latest ESC guidelines highlighted the usefulness of carotid ultrasound in the diagnosis of CAD. Ultrasound examination of peripheral arteries, such as the femoral arteries, is quite easy, quick and accurate, but other sites such as iliac and renal, although more demanding to examine, can also provide valuable information [9]. 

The present review aims to summarize the most important studies which analyzed the correlation between atherosclerosis detected by ultrasound examination in different vascular sites and the presence and severity of CAD, as well as the impact of the early detection of PAD on the outcomes of patients presenting CAD as well. 

## 2. Lower Extremity Arterial Disease and Coronary Artery Disease

In 2017, approximatively 202 million people were reported to be affected by lower extremity arterial disease (LEAD) worldwide, with almost 40 million of them living in Europe [10]. As the prevalence of LEAD increases with age, ranging from 1% to 3% in the fourth decade to >20% in the eighth decade [11], it is expected that this number is continuously increasing. Most of the patients are asymptomatic, detected either by a low ankle-brachial index (ABI) (<0.90) or pulse abolition [10]; however, asymptomatic patients are still highly underdiagnosed as ABI is not routinely measured. As numerous studies have demonstrated the high rate of coexistence of atherosclerotic disease in multiple vascular sites, it is fair to assume that an early diagnosis of LEAD could determine an early diagnosis of CAD as well, with possible further impact on these patients’ outcomes. 

In a study including 1734 patients of whom 1253 were diagnosed with CAD, the co-existence of carotid artery stenosis, renal artery stenosis and LEAD, respectively, was reported in 7%, 9% and 16% and, what is more, the extent and severity of CAD and that of other atherosclerotic lesions were significantly correlated [12]. In an important pooled analysis of 19,867 patients undergoing PCI, 1602 (8.1%) were previously diagnosed with LEAD. These patients had higher incidences of 7-day death (1.0% vs. 0.4%; *p* < 0.001) or myocardial infarction (MI) (6.8% vs. 5.6%; *p* = 0.047), 30-day death (1.7% vs. 0.7%; *p* < 0.001) or MI (7.4% vs. 6.1%; *p* = 0.05), with consistent results at mid-term follow up regarding death or MI at 6 months (4.2% vs. 1.5%; *p* < 0.001 and 9.1%, vs. 7.7%; *p* = 0.048), and 1-year death (5.0% vs. 2.1%; *p* < 0.001) [3]. Lamina C et al. prospectively examined 1325 patients, with 51.8% of men and 36.3% of women presenting at least one plaque in the carotid or femoral arteries. At 13-year follow up, the authors described an increase in risk for MI and cardiovascular and total mortality of 52, 70 and 45%, respectively, for each increase in the number of plaque-affected arteries (*p* < 0.0001) [4]. What is more, it has been shown that patients with complex peripheral artery disease often present complex coronary artery disease as well [13]. In another major trial, the PEGASUS-TIMI 54, 1143 patients, representing 5% of the total studied population, had known PAD as well as prior myocardial infarction. At 3-year follow up, these patients presented a significantly higher rate of major adverse cardiac events (MACE) (19.3% vs. 8.4%; *p* < 0.001), as well as adjusted 2-fold increased rates of all-cause death, CV death and stroke [14]. 

These results emphasize the importance of an early assessment of the presence, severity and complexity of lower limb atherosclerosis. The latest ESC Guidelines granted Doppler ultrasound a class I indication as a first-line imaging method to confirm LEAD lesions [10]. The use of ultrasound not only has the ability to identify hemodynamically significant stenosis, but can also provide a variety of information that can serve as predictive factors, as shown in different studies. 

The most commonly used ultrasound parameters for predicting cardiovascular disease by analyzing the lower limb arteries are the femoral intima-media thickness (fIMT) and the femoral artery plaque characterization. Kocyigit D et al. conducted a study which included 215 patients without documented cardiovascular disease who were scheduled for coronary computed tomographic angiography and in which IMT measurements and plaque assessment were performed at femoral and carotid arteries previously. All patients were followed up for a median of 2 years, and MACE were encountered in 4.19% of them. In these patients, fIMT was increased and surface irregularities and ulceration in femoral artery plaques were more common (*p* = 0.001) as well, thus suggesting that femoral ultrasound assessment may provide prognostic information for predicting MACE in specific populations [15]. The fIMT had a specificity of 60% and sensitivity of 70% as an independent predictive factor for obstructive CAD in another study that enrolled 184 patients undergoing coronary angiography [16]. A strong positive correlation between fIMT and the severity of CAD was also described by Kirhmajer MV in 180 patients who had undergone coronarography for symptomatic CAD. What is more, the authors reported significant positive correlations between this parameter and other traditional CV risk factors such as triglycerides (*p* = 0.017), body mass index (BMI; *p* = 0.036), male gender (*p* = 0.0000) and smoking (*p* = 0.028), suggesting that fIMT could represent a cardiovascular risk marker itself [17]. Sosnowski C et al. assessed the carotid and femoral IMT in 410 patients undergoing elective coronary arteriography, which was positive for CAD in 81% of the patients [18]. Interestingly, in this study fIMT was an independent predictor of a single-vessel disease, whilst femoral atherosclerotic plaque presence was associated with advanced CAD. As ultrasonography is known to be an operator-dependent imaging technique, several small studies have tested the reproducibility of fIMT as a prognostic parameter. Srámek A et al. reported good reproducibility and reliability in healthy subjects, but with lower reproducibility in patients with clinical atherosclerosis [19]. 

**Figure 1:**
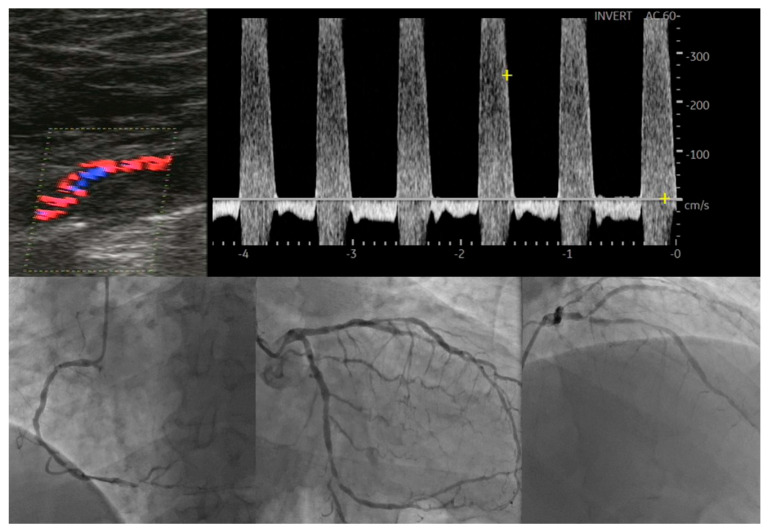
Correlation between lower extremity artery disease and coronary artery disease.

Several other ultrasonography parameters have been proposed as valuable tools in predicting the presence and severity of CAD in patients with asymptomatic LEAD. Yerly P et al. described a novel ultrasonographic score that sums the number of carotid and femoral arterial bifurcations with plaques, that is, the atherosclerosis burden score (ABS) [20]. By using ultrasonography, 203 patients undergoing coronary angiography were assessed and ABS was calculated, significantly outperforming common carotid IMT, carotid mean/maximal thickness and carotid/femoral plaque scores for the detection of CAD. Of great importance as well, this score was also more correlated with CAD extension (R = 0.55; *p* < 0.001). Santoro L et al. proposed a new ultrasonographic score, the ULLA (ultrasonographic lower limb atherosclerosis) score, that categorizes atherosclerotic lesions of the lower limbs at all stages of PAD. They evaluated the correlation of these ultrasonographic categories with CV risk profiles in 320 consecutive patients [21]. All arterial segments were examined for their parietal characteristics, in particular the presence of vessel wall calcifications and/or atherosclerotic plaque; flow velocity measurements were also obtained. Based on these characteristics, lesions in the proximal district were categorized into six groups, while lesions in the distal district were categorized into five groups. The authors reported a significant association of this severity score with the main CV risk factors, represented by age, male gender, cigarette smoking, arterial hypertension, diabetes, dyslipidemia, sedentary lifestyle, previous CV events and family history of CV disease. Figure 1 serves as an example of severe coronary artery disease found in a diabetic patient with bilateral lower limb ischemia that exhibited atypical angina, but with a positive stress test. 

As an abnormal ankle-brachial index is traditionally the most reliable non-invasive tool suggesting the presence of LEAD, several authors compared the performance of this variable with that of ultrasonography evaluation. For this purpose, Colledanchise KN et al. measured ankle-brachial index in 124 patients who underwent femoral ultrasound for the assessment of intima-media thickness, maximal plaque height and total plaque area, with 64% of them presenting angiography-confirmed CAD. In these patients, femoral ultrasound had a higher sensitivity (85%) than the ABI (25%) for ruling out significant CAD, but both tools presented similar capacity to detect LEAD [22]. Similar results were reported by Santoro L et al., who found ultrasonography to be a better tool for identifying all LEAD stages and predicting cardiovascular events [23]. Table 1 summarizes the main studies evaluating the role of LEAD assessment by ultrasonography in predicting the presence and severity of coronary artery disease.

All these results emphasize the important role of ultrasonography in identifying asymptomatic lower extremity arterial disease patients and, of great importance, in predicting the risk of concomitant occurrence of coronary artery disease in these patients. Although these studies reported encouraging results regarding the role of several parameters in predicting the coexistence of LEAD and CAD, it must be taken into consideration that most of the studies were conducted on small cohorts of patients, thus lacking the statistical power to formulate robust recommendations for clinical practice. What is more, while ultrasound assessment in general is by definition limited by inter-operator variability, the use of more complex parameters could lead to further potential biases. Keeping in mind these results, several important studies have been conducted in order to evaluate the role of ultrasonographic assessment of atherosclerotic lesions in other arterial sites in predicting the presence and severity of CAD.

## 3. Aortic Atherosclerosis and Coronary Artery Disease

**Figure 2:**
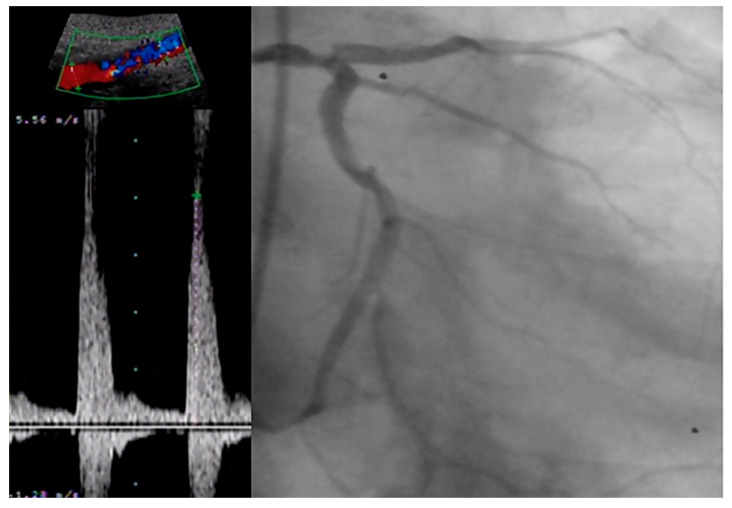
Correlation between severe distal aortic stenosis and coronary artery disease.

Although the association between abdominal aortic plaques and CAD has been hypothesized to exist, this correlation has not yet been clarified. Li W et al. conducted a prospective study enrolling 1667 consecutive patients undergoing coronary angiography, for which ultrasonography of the abdominal aorta was performed as well [24]. A total number of 1268 patients were identified with CAD, and in these patients the prevalence of abdominal aortic plaques was significantly higher (37.3% vs. 17%, *p* < 0.001). What is more, the authors found the presence of abdominal aortic plaques to be an independent predictor not only for the presence of CAD but for the severity as well, as the increase in the number of plaques was associated with an increase in the number of affected coronaries (*p* < 0.001; *p*-value for trend <0.001). Figure 2 illustrates the good correlation between a severe calcific stenosis of the distal aorta and a diffuse left main disease in a patient presenting with a chronic coronary syndrome. 

In a similar fashion, the strong association between CAD and thoracic atherosclerosis mainly determined by transesophageal echocardiography has been described in several studies [25,26]; however, there are limited data on the role of transthoracic ultrasound for predicting the presence and severity of coronary disease. In a prospective study using transesophageal echography, Kim HY et al. not only found a significant association between the presence of thoracic aorta plaques and the presence and severity of CAD, but also found the absence of thoracic aortic atherosclerosis to be a strong predictor of the absence of CAD [27]. Although several other studies with similar designs reported atherosclerosis of the thoracic aorta to be an independent predictor of long-term neurologic events and mortality [28], there are still conflicting data, as Meissner I et al. conducted a prospective study including 585 patients with complex (>4 mm thick or mobile debris) aortic atherosclerotic plaques, which were followed up for a median of five years, with no significantly increase in the number of cardiac or cerebrovascular events being described [29]. Therefore, larger studies are needed in order to confirm the role of aortic plaques determined by ultrasound as a predictive marker of future coronary events. 

## 4. Carotid Artery Disease and Coronary Artery Disease

Advances in non-invasive imaging together with a favorable anatomic location make the ultrasound evaluation of the carotid arteries an ideal technique for cardiovascular (CV) risk assessment and prediction of coronary artery disease (CAD) presence and severity.

While the 2019 ACC/AHA Guidelines on the Primary Prevention of Cardiovascular Disease mention only coronary artery calcium in the risk assessment of CV disease [30], the 2021 ESC Guidelines grant a II B indication for the use of carotid ultrasonography in the non-invasive evaluation of patients with suspected CAD [31].

**Figure 3:**
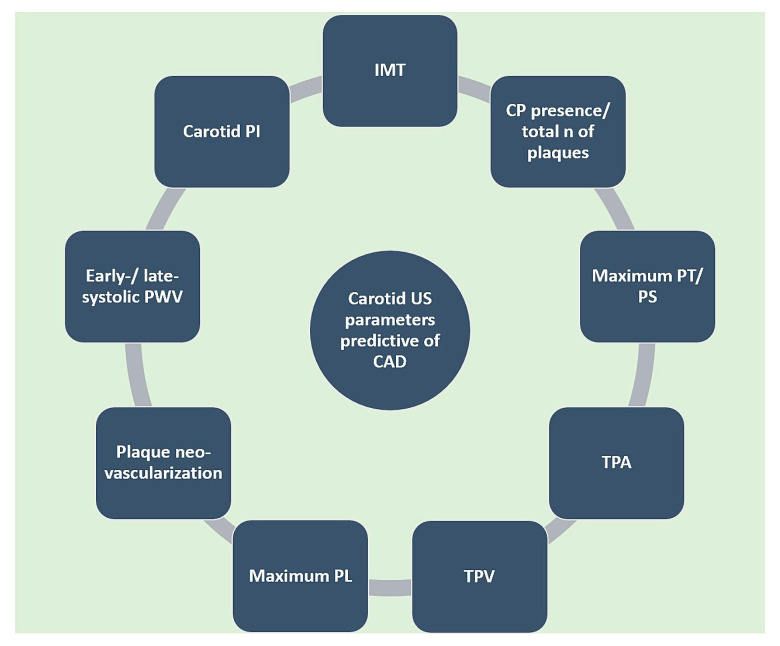
Carotid US-derived parameters predictive of CAD. CAD: coronary artery disease. CP: carotid plaque. IMT: intima-media thickness. N: number. PI: pulsatility index. PL: plaque length. PS: plaque score. PT: plaque thickness. PWV: pulse wave velocity. TPA: total plaque area. TPV: total plaque volume. US: ultrasound.

**Figure 4:**
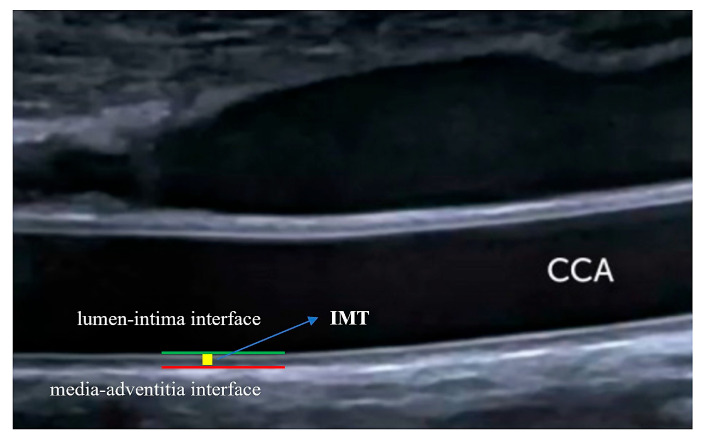
Intima-media thickness measurement. IMT is defined in the absence of carotid plaque as the distance between the lumen–intima and media–adventitia interfaces. Long-axis assessment of the far wall of the CCA is used. IMT: intima-media thickness. CCA: common carotid artery.

There are a number of ultrasound parameters used for the quantification of carotid atherosclerosis (Figure 3), with varying prognostic usefulness. Intima-media thickness (IMT) (Figure 4) has been shown to significantly predict CAD severity and extent, but only in the mid and distal and not proximal coronary segments [32]. However, a recent metanalysis of 89 studies demonstrated only a moderate correlation between IMT and degree of stenosis (r = 0.60; *p* < 0.001) and number of diseased vessels (r = 0.49; *p* < 0.001) [33]. It is noteworthy that integrating data from carotid and femoral arteries into an IMT score had a superior predictive power compared to individual IMT [34]. In a large-scale metanalysis including over 54,000 patients, Inaba et al. concluded that IMT had a similar diagnostic accuracy to carotid plaque in detecting CAD (AUC 0.74 vs. 0.76; *p* = 0.21 for relative DOR) but lower predictive power of future myocardial infarction (MI) events (AUC 0.61 vs. 0.64, relative DOR 1.35; 95%CI 1.1–1.82; *p* = 0.04) [35].

**Figure 5:**
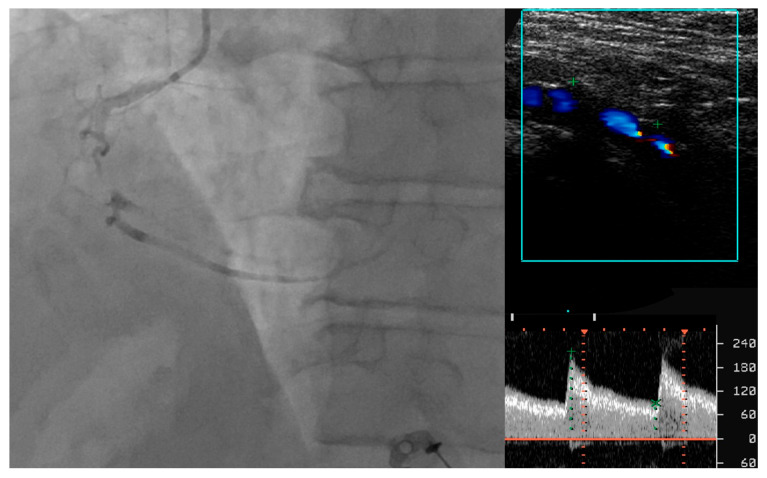
Correlation between carotid artery disease and coronary artery disease.

Adding to its increased value over IMT, the presence of carotid plaque in 433 patients with proven CAD was found to be predictive of cardiac death and hard major adverse cardiovascular events (MACE) (a composite of death, MI and stroke) at a mean follow up period of 54 months (HR 6.99, 95% CI 1.88–25.95, *p* = 0.004; HR 1.89, 95% CI 1.18–3.04, *p* = 0.008) [36]. Figure 5 illustrates the good correlation between a severe calcific stenosis of the right internal carotid artery and a subocclusive lesion found in the medium segment of the right coronary artery in a patient presenting with an acute coronary syndrome. 

Maximum plaque thickness and plaque score (PS), a sum of all plaque thickness measurements in both carotid arteries, seem to provide additional predictive value. In a study by Ikeda et al., PS was superior compared to IMT in predicting complex CAD, defined by an intermediate or high SINTAX score (OR 1.31, 95% CI: 1.23–1.39; *p* < 0.001; OR 1.24, 95% CI: 1.17–1.32; *p* < 0.001). Moreover, PS was the only independent predictor of the presence of CAD (OR 1.22, 95% CI: 1.14–1.31; *p* < 0.001) and complex CAD (OR 1.31, 95% CI: 1.20–1.43; *p* < 0.001) [37]. As it is simpler to perform, maximum plaque thickness was tested in a cohort of 6102 asymptomatic patients. Over a median follow-up period of 2.7 years, primary (CV death, MI or ischemic stroke) and secondary (all-cause death, MI, ischemic stroke, unstable angina or coronary revascularization) MACE were collected. Maximum plaque thickness was predictive of both primary and secondary MACE (HR 1.96, 95% CI 0.91–4.25, *p* = 0.015; HR 3.13, 95% CI 1.80–5.51, *p* < 0.001) in a similar fashion to carotid plaque burden, a more complex but cumbersome assessment tool [37].

Data from the Multi-Ethnic Study of Atherosclerosis spanning over 13 years and including subjects that were initially free of CV disease, but with established carotid plaques, showed that total plaque area was a strong predictor of coronary events (HR 1.23, 95% CI 1.11–1.36; *p* < 0.001) [38]. Similar results were observed for the carotid plaque score, a parameter consisting of the total number of arterial segments with a plaque (HR 1.33, 95% CI 1.18–1.49; *p* < 0.001) [39]. Wu et al. developed a parametric model including areas of maximum soft, hard and mixed carotid plaques, total number of plaques and clinical risk factors that provided a useful tool in detecting obstructive CAD [40].

Providing a more accurate atherosclerosis assessment, carotid total plaque volume was studied in conjunction with IMT and plaque area in order to investigate the link between plaque progression/regression and CV outcomes. Progression of plaque volume (median 27 mm^3^) significantly predicted CV events (*p* = 0001), even after adjustment for coronary risk factors (*p* = 0001) [41]. What is more, in a small prospective study a measured plaque volume above the threshold of 0.09 mL was predictive of significant CAD [42]. 

More recently, Tang et al. conducted a prospective study on 2149 patients in order to assess the relationship between carotid plaque length (CPL) and CAD, in comparison to other ultrasonography parameters (IMT and PS). The Gensini score (GS) was more strongly correlated with max CPL as compared to PS and mean IMT. In addition, max CPL was independently associated with CAD and high GS, even after multivariate analysis. Max CPL displayed significant sensitivity and negative predictive value (84.6 and 89.1%) for high GS at a cut-off of 6.3 mm [43].

Several other ultrasound measurements have been tested, with encouraging results. By means of a novel ultrafast ultrasound imaging technique, Li et al. calculated carotid early- and late-systolic pulse wave velocity as indices of wall stiffness. Both parameters were indicative of the presence of CAD (*p* < 0.001) and significantly correlated with the GS (β = 0.466, *p* < 0.001; β = 0.308, *p* < 0.001) [44]. The carotid pulsatility index was found to be an independent predictor of CV events at a specific threshold for each level of the carotid arterial tree. Interestingly, a high external carotid artery pulsatility index was associated with increased MACE rate as compared to the internal carotid artery and common carotid artery groups (HR 11.322, *p* = 0.001; HR 3.639, *p* = 0.012; HR 3.242, *p* = 0.042) [45,46].

In a single-center prospective study including stable patients referred for coronary angiography (CA), carotid ultrasound was performed in order to detect neovascularization, a marker of plaque vulnerability. A high neovascularization score was not only associated with (1.7 ± 0.5 vs. 1.3 ± 0.8; *p* < 0.0001) but predictive of significant CAD (sensitivity 92%; specificity 89%). At 30-day follow up, patients with a neovascularization score above the cut-off value of 1.25 experienced a higher proportion of CV events (*p* = 0.004) [47]. 

## 5. Visceral Artery Disease and Coronary Artery Disease

In most of the studies evaluating renal artery stenosis, the diagnosis was made by angiography rather than ultrasound. In a cross-sectional study including over 1700 patients, the relationship between systemic atherosclerotic disease on ultrasound and CAD was analyzed. The prevalence of renal artery stenosis was 9% in the overall CAD population and it increased with CAD severity (13% in patients with three-vessel disease, *p* < 0.0001). A strong correlation between CAD severity and the risk of renal artery stenosis as well as carotid and peripheral artery disease was found. The strongest independent predictors of renal stenosis were three-vessel disease (OR 4.79, 95% CI 2.32–9.89; *p* < 0.001), left main disease (OR 3.16, 95% CI 1.44–6.94; *p* = 0.004), carotid stenosis (OR 4.13, 95% CI 2.38–7.15; *p* < 0.001) and peripheral artery disease (OR 2.36, 95% CI 1.49–3.73; *p* < 0.001) [12]. In a smaller analysis, patients with renal artery stenosis had higher coronary calcium scores and required more revascularizations at the 1-year follow up as compared to the control group [48]. 

The renal resistive index (RI), renal pulsatility index (PI) and acceleration time (AT) are measurements reflective of intrarenal vasculature and are proven predictors of CV events [49]. In a cross-sectional study including 235 patients with acute coronary syndromes referred for CA, the relationship between the extent of CAD and renal Doppler parameters was evaluated. In patients with non-ST segment elevation MI, RI (β = 32.230, 95% CI 5343.15–2 × 10^24^; *p* = 0.008) and PI (β = −7.439, 95% CI 0.000–0.231; *p* = 0.015) were independent predictors of a moderate-to-high SYNTAX score, while, surprisingly, no predictors were found in the ST-segment elevation MI group [50]. Geraci et al. found that in patients with stable CAD PI was associated with the presence of mild atherosclerotic burden (*p* = 0.047), whereas this association was not identified in case of a higher GS (cut-off ≤30) [51]. What is more, an increased RI (driven mainly by lower end-diastolic velocities) is associated with a worse 24-month prognosis in a cohort of both stable and unstable patients referred for CA (OR 1.11 per 0.01, 95% CI 1.02–1.20; *p* = 0.02). There was impaired survival in patients with a pre-procedural RI above the threshold of 0.0645 (log-rank *p*  <  0.001) [52].

Renal artery stenosis does not seem to be associated with increased mortality in elective patients undergoing coronary artery bypass grafting, even at very long-term follow up [53,54]. In contrast, Aboyans et al. show that the RI is a strong predictor of both the postoperative development of acute kidney injury (OR 2.4, 95% CI 1.01–5.82; *p* = 0.0475) as well as the 30-day (OR 4.3, 95% CI 1.87–9.89; *p* = 0.0006) and mid-term (OR 5.4, 95% CI 2.14–13.82; *p* = 0.0004) mortality and CV events [53].

## 6. Cost-Effectiveness and Future Perspectives

As recent studies have published more and more information on the molecular mechanisms of PAD, there is constant interest in finding potential correlations between them and different imaging markers for maximizing their diagnostic and prognostic potential. As a result, several new imaging technologies have the potential to overcome the current challenges in the early diagnosis of asymptomatic peripheral disease. For example, dual-energy computed tomography (CT) angiography, also known as spectral CT, uses two separate X-ray energy spectra and offers much more detailed tissue imaging, thus detecting significant stenoses with higher sensitivity. Another novel technique, photon counting CT (PCCT), has also been shown to possess multiple advantages over conventional CT angiography, thus improving the diagnosis of PAD. Important advances have also been reported in the invasive diagnosis and treatment of PAD. In the past two decades, the feasibility of robotic peripheral vascular interventions and that of diagnostic angiography with active guide catheter control were reported with promising achievements. What is more, the use of intravascular ultrasound and optical coherence tomography, which are well-established coronary imaging modalities, have now emerged for lower extremity endovascular imaging as well, providing higher-resolution imaging with two- and three-dimensional images and thus having the potential to optimize the outcomes of endovascular interventions. However, future studies are needed in order to formulate conclusions or recommendations.

Another important future direction in the management of patients with peripheral artery disease is represented by the Vascular Team concept. In the latest European guidelines, the management of patients with peripheral artery disease by a multidisciplinary Vascular Team was granted a class I, level of evidence C recommendation. This recommendation mainly emerges from the common pathophysiological mechanisms linking PAD and CAD. Although it is not fully understood why plaque formation has such a heterogeneous distribution, there are two possible factors that could explain the link between PAD and CAD: first, the similarities in the anatomy of large arteries (i.e., a single layer of endothelial cells); second, the similarities in the inflammatory signaling pathways that are activated, thus allowing the fatty streak formation that represents the first sign of atherosclerosis, characterized by a substantial accumulation of lipids both within the cells and the extracellular media [55,56,57]. 

Although multisite artery disease (MSAD) is common in patients with atherosclerotic involvement in one vascular bed and is associated with worse outcomes, and even though multiple studies have highlighted the predictive role of ultrasound assessment in identifying CAD patients, screening for asymptomatic disease in additional vascular sites has not been proven to improve prognosis [10,58]. However, the European guidelines recommend the clinical assessment of potential multisite artery disease, with further tests, including ultrasound, being recommended if clinical suspicion is present. What is more, the documents grant duplex ultrasound a class I recommendation for the diagnosis of renal artery disease, carotid artery disease and LEAD. It must be noted, however, that the questionable benefit of systematic screening for asymptomatic MSAD in patients with known atherosclerotic disease was mainly concluded from the AMERICA trial, which has several limitations and a relatively small population; therefore, the potential of the ultrasound assessment of asymptomatic patients for identifying CAD patients and improving their outcomes must still be studied.

In light of the above, in the last few years, technological developments in the surgical field have been rapid and are continuously evolving. One of the most revolutionary breakthroughs was the introduction of the “Internet of Things” (IoT) concept within surgical practice. In the field of PAD, this concept could find its usefulness in various forms, ranging from awe-inspiring telesurgical procedures to complex AI machine learning applications that aid in medical decision making [59]; however, further studies are needed. 

## 7. Conclusions

Atherosclerosis is a generalized condition and its simultaneous presence in the coronary arteries and other vascular sites is not uncommon. As clinically manifested peripheral artery disease has been reported to worsen the outcomes of ischemic heart disease, the detection of subclinical atherosclerosis could be a useful tool in predicting the risk of CAD. Ultrasonography has proven to be a reliable method for this detection. Several US parameters have been proposed in the assessment of PAD, with varying prognostic usefulness depending on disease location. 

## Figures and Tables

**Table 1:** Summary of the main studies evaluating lower extremity arterial disease by ultrasound in relation with coronary artery disease.

Study	Results
Saw J et al. [3]	Patients undergoing PCI and associated LEAD vs. without LEAD had increased 6-month death and MI rate (4.2 vs. 1.5%, *p* < 0.00; 9.1 vs. 7.7%, *p* = 0.048)
Lamina C et al. [4]	A 52, 70 and 45% increased risk of MI, CV and total death for each additional plaque-affected femoral/carotid artery (*p* < 0.0001)
Imori Y et al. [12]	Significant correlation between LEAD and the extent of CAD (*p* < 0.0001); 27 and 30% prevalence of LEAD in patients with three-vessel disease and left main disease, respectively
Aykan AC et al. [13]	Correlation between complex LEAD and complex CAD (β = 0.282, 95% CI 0.431–0.782; *p* < 0.001)
PEGASUS-TIMI 54 trial [14]	Patients with prior MI and LEAD vs. without LEAD had higher 3-year MACE (19.3 vs. 8.4%; *p* < 0.001), CV death (HR 1.84, 95% CI 1.16–2.94; *p* = 0.0102), total death (HR 2.05, 95% CI 1.43–2.94; *p* < 0.001) and stroke (HR 2.31, 95% CI 1.26–4.25; *p* = 0.0071) rates
Kocyigit D et al. [15]	Patients scheduled for coronary CT angiography who experienced MACE at 2-year follow up had increased fIMT (*p* = 0.015), femoral plaque irregularities and ulcerations (*p* = 0.001)
Kafetzakis A et al. [16]	fIMT was predictive of obstructive CAD (Sp 60%, Se 70%)
Kirhmajer MV et al. [17]	Correlation between fIMT and presence of CAD (*p* = 0.0000), severity of CAD (*p* = 0.0000) and other CV risk factors
Sosnowski C et al. [18]	fIMT predicts single-vessel CAD (OR 5.0, 95% CI 1.4–18.4) while the presence of femoral plaque predicts multi-vessel CAD (OR 1.4, 95% CI 1.0–2.0)
Srámek A et al. [19]	fIMT has good reproducibility and reliability in healthy subjects (variability 5.5%) but lower reproducibility in patients with clinical atherosclerosis (variability 17.5%)
Yerly P et al. [20]	Atherosclerosis burden score (sum of carotid and femoral arterial bifurcations with plaques) is superior to femoral plaque score in detecting CAD (AUC 0.79, 95% CI 0.73–0.86; *p* = 0.027) and predicting CAD extension (R = 0.55; *p* < 0.001)
Santoro L et al. [21]	The US lower limb atherosclerosis score (which facilitates the categorization of atherosclerotic lesions of the lower limbs in all stages of LEAD) is associated with CV risk factors, previous CV events (*p* = 0.01) and family history of CV disease (*p* = 0.05)
Colledanchise KN et al. [22]	Femoral US had a higher Se (85%) than the ABI (25%) for ruling out significant CAD, but similar capacity to detect LEAD; femoral total plaque area had the strongest association with CAD (rho = 0.37; *p* < 0.0001)
Santoro L et al. [23]	Femoral US is superior to ABI in predicting LEAD at all stages, as well as CV events

ABI: ankle-brachial index. CAD: coronary artery disease. CV: cardiovascular. fIMT: femoral intima-media index. LEAD: lower extremity arterial disease. MACE: major adverse cardiovascular events. MI: myocardial infarction. PCI: percutaneous coronary intervention. Se: sensibility. Sp: specificity. US: ultrasound.
